# Self-Diffusion in Simple Liquids as a Random Walk Process

**DOI:** 10.3390/molecules26247499

**Published:** 2021-12-11

**Authors:** Sergey A. Khrapak

**Affiliations:** Joint Institute for High Temperatures, Russian Academy of Sciences, 125412 Moscow, Russia; sergey.khrapak@gmx.de

**Keywords:** self-diffusion in liquids, transport properties of liquids, random walk process, viscosity of liquids, one-component plasma, collective motion in liquids

## Abstract

It is demonstrated that self-diffusion in dense liquids can be considered a random walk process; its characteristic length and time scales are identified. This represents an alternative to the often assumed hopping mechanism of diffusion in the liquid state. The approach is illustrated using the one-component plasma model.

## 1. Introduction

About 40 years ago, Robert Zwanzig published an influential paper on the relation between self-diffusion and viscosity of liquids (Stokes–Einstein relation) [1]. The purpose of the present paper is to demonstrate that the dynamical picture behind Zwanzig’s result is equivalent to a random walk process, with well defined length and time scales. It is also demonstrated that a theoretical prediction for the numerical factor relating the self-diffusion and viscosity coefficients, in the form of the Stokes–Einstein relation, is quite sensitive to concrete assumptions about the liquid collective mode spectrum. The results provide a consistent picture of the diffusion mechanism in dense liquids with soft isotropic pairwise interactions.

## 2. Results

### 2.1. Diffusion as Random Walk

Self-diffusion usually describes the displacement of a test particle immersed in a medium with no external gradients. A canonical example is the Brownian motion, representing a random motion of macroscopic particles suspended in a liquid or a gas. Here, we are interested in atomic scales and, hence, consider displacements of a labeled atom in a fluid of unlabeled, but otherwise identical, atoms. If this motion can be considered a random walk process, then the diffusion coefficient in three spatial dimensions can be defined as [2]

(1) $$D=\frac{1}{6}\frac{\langle r^{2}\rangle }{\tau },$$

where *r* is an actual (variable) length of the random walk, τ is the time scale, and we focus on sufficiently long times (t≫τ). Consider first an ideal gas as an appropriate example. The atoms move freely between pairwise collisions. If the distribution of free paths between collisions follows the $e^{-r/\lambda }/\lambda$ scaling, then 〈r〉=λ and $\langle r^{2}\rangle =2\lambda^{2}$, where λ is the mean free path [2]. Combining this with the relation for the average atom velocity 〈v〉=λ/τ, we recover the elementary kinetic formula for the diffusion coefficient of an ideal gas

(2) $$D=\frac{1}{3}\langle v\rangle \lambda .$$

The dynamical picture is very different in liquids and this simple consideration clearly does not apply. The very concept of random walk, however, remains relevant, although characteristic length and time scales associated with a random walk process in liquids are very different from those in gases.

Below, the model proposed by Zwanzig [1] to describe relations between the self-diffusion and shear viscosity coefficients of liquids, is discussed in some detail. In doing so, we naturally repeat some arguments and formulas from Zwanzig’s original work and later publications (for instance, from a recent paper by the present author [3]). The emphasis is, however, not on the Stokes–Einstein relation per se, but rather on the possibility of presenting self-diffusion as a random walk process, and on defining the associated length and time scales. The emerging picture represents an alternative to the often assumed hopping mechanism of diffusion in the liquid state.

Zwanzig’s approach is based on the assumption that atoms in liquids exhibit solid-like oscillations about temporary equilibrium positions corresponding to a local minimum on the system’s potential energy surface [2,4]. These positions do not form a regular lattice like in crystalline solids. They are also not fixed, and change (or drift) with time (this is why liquids can flow), but on much longer time scales. Local configurations of atoms are preserved for some time until a fluctuation in the kinetic energy allows rearranging the positions of some of the atoms towards a new local minimum in the multidimensional potential energy surface. The waiting time distribution of the rearrangements scales as exp(−t/τ)/τ, where τ is a lifetime. Atomic motions after the rearrangements are uncorrelated with motions before rearrangements [1].

Within this ansatz, a simplest reasonable approximation for the velocity autocorrelation function of an atom *j* is

(3) $$Z_{j}\left(t\right)≃\left(\frac{T}{m}\right)\cos\left(\omega_{j}t\right)e^{-t/\tau },$$

corresponding to a time dependence of a damped harmonic oscillator. Here, *T* is the temperature in energy units, *m* is the atomic mass, and $\omega_{j}$ is an effective vibrational frequency. The self-diffusion coefficient *D* is given by the Green–Kubo formula

(4) $$D=\frac{1}{N}\int_{0}^{\infty }\sum_{j}Z_{j}\left(t\right)dt.$$

Zwanzig then assumed that vibrational frequencies $\omega_{j}$ are related to the collective mode spectrum and performs averaging over collective modes. After the evaluation of the time integral, this yields

(5) $$D=\frac{T}{3mN}\sum_{k}\frac{\tau }{1+\omega_{k}^{2}\tau^{2}},$$

where the summation runs over 3N normal mode frequencies. The dynamical picture used by Zwanzig makes sense only if the waiting time τ is much longer than the inverse characteristic frequency of the solid-like oscillations. In this case, we can rewrite Equation (5) as

(6) $$D=\frac{T}{m\tau }\langle \frac{1}{\omega^{2}}\rangle ,$$

where the conventional definition of averaging, $\langle \omega^{-2}\rangle =\left(1/3N\right)\sum_{k}\omega_{k}^{-2}$ has been used.

Equation (6) allows for a simple physical interpretation. It represents a diffusion coefficient for a random walk process, Equation (1). The length scale of this process is identified as

(7) $$\langle r^{2}\rangle =\frac{6T}{m}\langle \frac{1}{\omega^{2}}\rangle ,$$

which is twice the mean-square displacement of an atom from its local equilibrium position due to solid-like vibrations [5]. The coefficient of two appears, because the initial atom position is not at the local equilibrium, but randomly distributed with the same properties as the final one (after the waiting time τ). The characteristic time scale of the random walk process is just the waiting time τ. Moreover, this waiting time should be associated with the Maxwellian shear relaxation time [2,6]

(8) $$\tau_{M}=\frac{\eta }{G_{\infty }}=\frac{\eta }{mnc_{t}^{2}},$$

where η is the shear viscosity coefficient, $G_{\infty }$ is the infinite frequency (instantaneous) shear modulus, *n* is the density, and $c_{t}$ is the transverse sound velocity.

Thus, self-diffusion in the liquid state can be viewed as a random walk due to atomic vibrations around temporary equilibrium positions over time scales associated with rearrangements of these equilibrium positions. In this paradigm, consecutive changes of temporary equilibrium positions (jumps of liquid configurations between two neighboring local minima of the multidimensional potential energy surface in Zwanzig’s terminology) are relatively small, much smaller than the vibrational amplitude. Hopping events with displacement amplitudes of the order of interatomic separation may be present, but they are relatively rare and do not contribute to the diffusion process. This picture is very different from the widely accepted hopping mechanism of self-diffusion in liquids. Previously, the concept of random walk was suggested in the context of molecular and atomic motion in water and liquid argon [7]. Here, we provide a more quantitative basis for this treatment.

Substituting Equation (8) into Equation (6), we obtain a relation between the self-diffusion and viscosity coefficients in the form of the Stokes–Einstein (SE) relation,

(9) $$D\eta \left(\frac{\Delta }{T}\right)=\frac{c_{t}^{2}}{\Delta^{2}}\langle \frac{1}{\omega^{2}}\rangle =\alpha_{\mathrm{SE}},$$

where $\Delta =n^{-1/3}$ is the mean interatomic separation and $\alpha_{\mathrm{SE}}$ is the SE coefficient.

Formula (9) particularly emphasizes the relation between the liquid transport and collective mode properties. Since the exact distribution of frequencies is generally not available, Zwanzig originally used a Debye approximation, characterized by one longitudinal and two transverse modes with acoustic dispersion. The sum over frequencies can be converted to an integral over *k* using the standard procedure $\sum_{k}\to V\int dk/{\left(2\pi \right)}^{3}$, where *V* is the volume. This yields

(10) $$\langle \frac{1}{\omega^{2}}\rangle =\frac{1}{6\pi^{2}n}\int_{0}^{k_{\max}}k^{2}dk\left(\frac{1}{\omega_{l}^{2}}+\frac{2}{\omega_{t}^{2}}\right),$$

where the cutoff $k_{\max}={\left(6\pi^{2}n\right)}^{1/3}$ is chosen to provide *n* modes in each branch of the spectrum. This ensures that the averaging procedure applied to a quantity that does not depend on *k* does not change its value. Substituting $\omega_{l}=c_{l}k$ and $\omega_{t}=c_{t}k$ into Equation (10) we arrive at

(11) $$\alpha_{\mathrm{SE}}=\frac{2}{{\left(6\pi^{2}\right)}^{2/3}}\left(1+\frac{c_{t}^{2}}{2c_{l}^{2}}\right)≃0.13\left(1+\frac{c_{t}^{2}}{2c_{l}^{2}}\right).$$

This essentially coincides with Zwanzig’s original result, except he expressed the SE coefficient in terms of the longitudinal and shear viscosity $\alpha_{\mathrm{SE}}≃0.13\left(1+\eta /2\eta_{l}\right)$. The equivalence was pointed out in Reference [6]. Note that since the sound velocity ratio $c_{t}/c_{l}$ is confined in the range from 0 to $\sqrt{3}/2$, the coefficient $\alpha_{\mathrm{SE}}$ can vary only between ≃0.13 and ≃0.18 [1,6]. Possible relations between the viscosity and thermal conductivity coefficients of dense fluids that can complement the SE relations of Equations (9) and (11) have been discussed recently [8].

An important time scale of a liquid state is a structure relaxation time. This can be defined as an average time it takes an atom to move the average interatomic distance Δ (sometimes it is referred to as the Frenkel relaxation time [9,10,11]). Taking into account diffusive atomic motions, we can write $\tau_{R}=\Delta^{2}/6D$. From Equation (1), we immediately get

(12) $$\tau_{R}=\frac{\Delta^{2}}{\langle r^{2}\rangle }\tau_{M}.$$

This implies that $\tau_{R}/\tau_{M}\gg 1$. The time scale ratio $\tau_{R}/\tau_{M}$ has a maximum at melting conditions, where, according to the Lindemann melting criterion $\Delta^{2}/\langle r^{2}\rangle ∼100$ [5,12]. This picture is consistent with the results from numerical simulations (see, e.g., Figure 3 from Reference [11]). Thus, there is a huge separation between the structure relaxation and individual atom dynamical relaxation time scales.

### 2.2. Relation to Collective Modes Properties

Despite the simplifications involved, the predictive power of Zwanzig’s model is quite impressive. Although the model does not allow making independent theoretical predictions of viscosity and self-diffusion coefficients, its prediction of the product, in the form of Equation (9), is highly accurate in some vicinity of the liquid–solid phase transition of many simple liquids [6,13,14]. Moreover, the coefficient $\alpha_{\mathrm{SE}}$ can be correlated with the potential softness (via the ratio of the sound velocities), as the model predicts [6]. Some of the assumptions, such as the effect of the waiting time distribution, were critically examined in Reference [15]. In particular, it was demonstrated that the SE relation of the form (9) is not obeyed if the distribution of waiting times is not exponential. In this section, we address another interesting question: how sensitive is the value of $\alpha_{\mathrm{SE}}$ to the assumptions about liquid collective mode properties?

To be specific, we consider a model one-component plasma (OCP) system. The OCP fluid is chosen for the following three main reasons: (i) vibrational (caging) motion is most pronounced due to extremely soft and long-ranged character of the interaction potential [16,17]; (ii) Zwanzig’s original derivation is not directly applicable to the OCP case, because the longitudinal mode is not acoustic (but plasmon) and, thus, it is a good opportunity to examine how the model should be modified in this case; (iii) collective modes in the OCP system are well studied and understood (for example, simple analytical expressions for the long-wavelength dispersion relations are available, see Appendix A).

The OCP model is an idealized system of mobile point charges immersed in a neutralizing fixed background of opposite charge (e.g., ions in the immobile background of electrons or vice versa) [18,19,20,21,22,23,24]. From the fundamental point of view, OCP is characterized by a very soft and long-ranged Coulomb interaction potential, $\phi \left(r\right)=q^{2}/r$, where *q* is the electric charge. The particle–particle correlations and thermodynamic properties of the OCP are characterized by a single dimensionless coupling parameter $\Gamma =q^{2}/aT$, where $a={\left(4\pi n/3\right)}^{-1/3}$ is the Wigner–Seitz radius. At Γ≳1, the OCP is strongly coupled, and this is where it exhibits properties characteristic of a fluid phase (a body centered cubic phase becomes thermodynamically stable at Γ≳174, as the comparison of fluid and solid Helmholtz free energies predicts [22,25,26]). Dynamical scales of the OCP are usually expressed by the plasma frequency $\omega_{p}=\sqrt{4\pi q^{2}n/m}$. For example, the Einstein frequency is $\Omega_{E}^{2}\equiv \langle \omega^{2}\rangle =\omega_{p}^{2}/3$. The transverse sound velocity at strong coupling is $c_{t}^{2}=\left(3/100\pi \right){\left(4\pi /3\right)}^{1/3}\omega_{p}^{2}\Delta^{2}≃0.015\omega_{p}^{2}\Delta^{2}$ [27].

**Figure 1:**
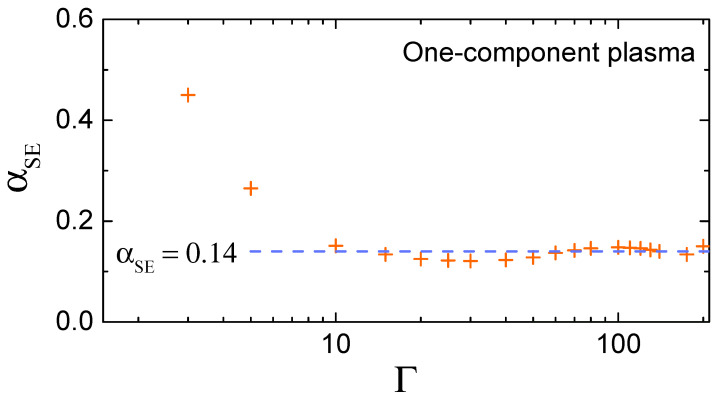
(Color online) Stokes–Einstein parameter $\alpha_{\mathrm{SE}}$ as a function of the coupling parameter Γ for a OCP fluid. The symbols correspond to MD simulation results from Refs. [28,29]. The dashed line shows a strong coupling asymptote $\alpha_{\mathrm{SE}}≃0.14$.

From extensive molecular dynamics simulations, it is known that the SE relation is satisfied to a very high accuracy in a strongly coupled OCP fluid with $\alpha_{\mathrm{SE}}≃0.14\pm 0.01$ at Γ≳50 [14,28,29]. Figure 1 demonstrates that $\alpha_{\mathrm{SE}}$ approaches the strongly coupled asymptote already at Γ≃10. Note that the OCP value of the SE coefficient is not truly universal, but rather representative for soft long-ranged pairwise interactions, in which case the transverse-to-longitudinal sound velocity ratio is small [see Equation (11)]. For example, the same value (≃0.14) is reached in weakly screened Coulomb (Yukawa) fluids, while for Lennard-Jones fluids it increases to $\alpha_{\mathrm{SE}}≃0.15$ and further to $\alpha_{\mathrm{SE}}≃0.17$ in hard-sphere fluids [14].

Now, we examine the sensitivity of the theoretical value of the SE coefficient $\alpha_{\mathrm{SE}}$ to concrete assumptions about the collective excitation spectrum. We start with the simplest approximation that all atoms are oscillating with the same Einstein frequency $\Omega_{E}$ (known as the Einstein model in the solid state physics). This approximation results in $\alpha_{\mathrm{SE}}≃0.046$, which is too low compared to the actual value from MD simulations (see Figure 1).

As a next level of approximation a Debye-like vibrational density of states (VDOS), $g\left(\omega \right)\propto \omega^{2}$ is assumed (averaging is performed using a standard definition $\langle \omega^{\ell }\rangle =\int \omega^{\ell }g\left(\omega \right)d\omega /\left(\int g\left(\omega \right)d\omega \right)$). Using the cutoff Debye frequency $\omega_{D}$ and requesting that $\langle \omega^{2}\rangle =\Omega_{E}^{2}$ we arrive at $\langle \omega^{-2}\rangle =9/5\Omega_{E}^{2}$. This yields $\alpha_{\mathrm{SE}}≃0.083$, which is somewhat better, but still considerably smaller than the actual result.

The most accurate theoretical estimate would be obtained if the exact VDOS were known. However, this is not the case. Nevertheless, accurate knowledge of the real dispersion relations for the longitudinal and transverse modes can be already quite useful. We make use of simple expressions based on the quasi-localized charge approximation (QLCA) [30] combined with the excluded cavity model for the radial distribution function [27]. The corresponding expressions for $\omega_{l}\left(k\right)$ and $\omega_{t}\left(k\right)$ are provided in the Appendix A. Substituting these in Equation (10), we have obtained $\langle \omega_{p}^{2}/\omega^{2}\rangle ≃9.76$ and $\alpha_{\mathrm{SE}}≃0.150$. This is very close to the exact result from MD simulations, as expected. Note that the exact result $\langle \omega^{2}/\omega_{p}^{2}\rangle =1/3$ is reproduced by construction.

The last demonstration uses a heuristic VDOS of the form

(13) $$g\left(\omega \right)=A\omega^{2}\exp\left(-B\omega^{2}\right),$$

which reproduces the Debye model at low frequencies and implements the Gaussian cutoff at high ω. This form was inspired by the observation that the functional form $g\left(\omega \right)=2\alpha \omega e^{-\alpha \omega^{2}}$ can fit the numerically obtained VDOS of Lennard-Jones liquids reasonably well [31]. We just substituted the linear scaling at low frequencies with the quadratic one to make the integral converging. This is clearly not a valid physical argument, but we use it here merely for illustrative purposes. The two normalization conditions yield

(14) $$A=\frac{4}{\sqrt{\pi }}{\left(\frac{3}{2\Omega_{E}^{2}}\right)}^{3/2},\;\;B=\frac{3}{2\Omega_{E}^{2}}.$$

Application of this VDOS results in $\alpha_{\mathrm{SE}}≃0.139$, which almost coincides with the exact MD result. Thus, implementation of the Gaussian cutoff to the Debye-like VDOS improves the situation considerably.

It should be noted that very long wavelengths and low frequency parts of the spectra are not relevant for the present consideration, because dynamics on time scales shorter than the relaxation time $\tau_{M}$ is considered. However, since $\omega \tau_{M}\gg 1$ needs to be satisfied, this corresponds to only a small part of the entire spectrum, and we therefore included low frequencies for simplicity, similar to what Zwanzig did originally [1]. This also allows us to disregard the effects associated with the *k*-gap in the dispersion relation of the transverse mode, an important property of liquid dynamics [32,33,34,35,36,37].

## 3. Discussion and Conclusions

While transport phenomena in gaseous and solid phases can be well described at the quantitative level, transport in liquids is still much less understood, even at the qualitative level. Here, we have demonstrated that self-diffusion in dense liquids can be described as a random walk process with well defined time and length scales. The length scale is related to the amplitude of solid-like vibrations around local temporary equilibrium positions. The time scale is set by the Maxwellian shear relaxation time. This dynamical picture results in the Stokes–Einstein relation between the coefficients of self-diffusion and viscosity, which is satisfied in many simple liquids. Importantly, the hoping mechanism of atomic diffusion in liquids is irrelevant in this picture of microscopic atomic dynamics.

The dynamical picture involved requires that the atomic motion be dominated by fast solid-like oscillations around the local equilibrium positions. This limits the model applicability to regions on the phase diagram located not too far from the liquid–solid phase transition (high densities and low temperatures). Additionally, it applies to sufficiently soft interaction potentials with pronounced oscillation dynamics. In the hard sphere interaction limit, this model is clearly inadequate (although SE relation is still satisfied even in this limit [14]).

Finally, we have demonstrated that a theoretically obtained numerical factor in the SE relation is sensitive to concrete assumptions about the liquid collective modes properties. This highlights the necessity of accurate knowledge of the vibrational density of states and dispersion relations in liquids.

## Data Availability

Not applicable as no new date were created or analyzed in this study.

## Abbreviations

The following abbreviations are used in this manuscript:

SE relation	Stokes–Einstein relation
OCP	one-component plasma
VDOS	vibrational density of states
QLCA	quasi-localized charge approximation

## Appendix A. Dispersion Relations of a Strongly Coupled OCP Fluid

**Table A1:** Averaged frequencies of a strongly coupled OCP fluid obtained with the help of dispersion relations (A1) and (A2).

$\langle \omega^{2}/\omega_{p}^{2}\rangle$	$\langle \omega /\omega_{p}\rangle$	$\langle \;\ln\omega /\omega_{p}\rangle$	$\langle \omega_{p}/\omega \rangle$	$\langle \omega_{p}^{2}/\omega^{2}\rangle$
$\frac{1}{3}$	0.514	−0.8023	2.5856	9.7623

Combining the QLCA model with a simple excluded cavity approximation for the radial distribution function, the following analytical expressions for the longitudinal and transverse dispersion relations in OCP fluids have been derived [27]

(A1) $$\omega_{l}^{2}=\omega_{p}^{2}\left(\frac{1}{3}-\frac{2\cos Rq}{R^{2}q^{2}}+\frac{2\sin Rq}{R^{3}q^{3}}\right)$$

and

(A2) $$\omega_{t}^{2}=\omega_{p}^{2}\left(\frac{1}{3}+\frac{\cos Rq}{R^{2}q^{2}}-\frac{\sin Rq}{R^{3}q^{3}}\right),$$

where q=ka is the reduced wave-number and *R* is the reduced excluded cavity radius. In the strongly coupled OCP regime, we have $R=\sqrt{6/5}≃1.09545$. Expressions (A1) and (A2) are rather accurate in the long-wavelength regime [38,39,40], except the existence of *k*-gap in the transverse mode is not accounted for [36]. Expressions (A1) and (A2) can be used to perform averaging over collective mode frequencies. We performed averaging of several frequency-related quantities and provide them in Table A1 for completeness.

The result for $\langle \omega^{2}/\omega_{p}^{2}\rangle \equiv 1/3$ is exact by virtue of Equations (A1) and (A2). The quantity $\langle \omega_{p}^{2}/\omega^{2}\rangle$ is used here to estimate the SE coefficient. The quantity $\langle \omega /\omega_{p}\rangle$ emerges in the vibrational model of thermal conductivity of simple fluids [3,41]. The quantity $\langle \;\ln\omega /\omega_{p}\rangle$ emerges in a variant of the cell theory of liquid entropy [42].
